# A collaborative cervical precancer screening strategy with concurrent HPV genotyping and visual inspection using alumni of a training centre across Ghana: The Rotary ‘Protect Your Pearl’ initiative

**DOI:** 10.1371/journal.pone.0350573

**Published:** 2026-06-26

**Authors:** Kofi Effah, Dorothy Letitia Ametefe, Joseph Emmanuel Amuah, Nana Owusu Mensah Essel, Maxwell Afetor, Annita Edinam Dugbazah, Emmanuel Deho, Seyram Kemawor, Stephen Danyo, Edna Sesenu, Georgina Tay, Yohane Teye Kitcher, Isaac Gedzah, Isaac Williams, Gifty Belinda Klutsey, Emmanuel Timmy Donkoh

**Affiliations:** 1 Cervical Cancer Prevention and Training Centre, Catholic Hospital, Battor, Ghana; 2 Rotary Club of Accra-East, Accra, Ghana; 3 School of Epidemiology and Public Health, Faculty of Medicine, University of Ottawa, Ottawa, Ontario, Canada; 4 Department of Emergency Medicine, College of Health Sciences, Faculty of Medicine and Dentistry, University of Alberta, Edmonton, Alberta, Canada; 5 Department of Epidemiology and Biostatistics, Fred N. Binka School of Public Health, University of Health and Allied Sciences, Ho, Volta Region, Ghana; 6 Volta Regional Health Directorate, Ghana Health Service, Ho, Volta Region, Ghana; 7 Screen and Treat Research Group, Centre for Research in Applied Biology, School of Sciences, University of Energy and Natural Resources, Sunyani, Ghana; Sefako Makgatho Health Sciences University, SOUTH AFRICA

## Abstract

**Background:**

Cervical cancer is a leading cause of cancer mortality among Ghanaian women, yet screening uptake is under 5%. The Cervical Cancer Prevention and Training Centre (CCPTC) partnered with Rotary Clubs across the country to implement the first-ever nationally representative cervical precancer screening project and to demonstrate the feasibility of an integrated nationwide screening program.

**Methods:**

We conducted a cross-sectional analysis of 1,636 asymptomatic women aged 25 years and above screened at 29 government and private facilities across all 16 regions of Ghana (January–February 2025). Eligible women underwent concurrent hr-HPV genotyping (Sansure MA-6000 platform) and VIA by CCPTC-trained alumni, with immediate thermal ablation for eligible VIA-positive lesions (TZ type 1 or 2). Multivariable logistic regression (backward stepwise elimination, P < 0.25 retention threshold) identified factors associated with hr-HPV positivity and VIA positivity. Analyses were performed in Stata v17.0.

**Results:**

Among 1,636 women, the overall hr-HPV prevalence was 26·6% (95% CI, 24·5–28·8) and the VIA ‘positivity’ was 4·0% (95% CI, 3·1–5·0). Predominant genotypes were HPV52 (5·3%), HPV58 (4·4%), and HPV51 (3·6%); HPV16 and HPV18 together accounted for <5% of infections. Independent factors associated with hr-HPV infection were HIV infection (aOR=5·77; 95% CI, 2·07–16·13, P = 0.001) and having a steady partner (aOR=2·02; 95% CI, 1·22–3·36, P = 0.006); being married/cohabiting (aOR=0·51; 95% CI, 0·38–0·69, P < 0.001) or widowed (aOR=0·43; 95% CI, 0·23–0·82, P = 0.011), and prior screening (aOR=0·67; 95% CI, 0·48–0·92, P = 0.014) were protective. VIA ‘positivity’ was independently associated with HIV infection (aOR 7.49, 95% CI 1.99–28.19, P = 0.003). Regional hr-HPV prevalence varied from 10·0% to 39·2%. Thirty-five percent of VIA-positive women received same-visit thermal ablation.

**Conclusion:**

This decentralized alumni-driven model integrating off-site HPV testing, task-shifted VIA, and immediate thermal ablation proved operationally feasible across Ghana’s diverse health system and revealed a substantial hr-HPV burden. The approach offers a scalable blueprint for national cervical cancer control and informs Ghana’s transition toward HPV-based screening.

## Introduction

Cervical cancer (CC) is a leading cause of cancer-related mortality among women worldwide, despite being largely preventable through effective human papillomavirus (HPV) vaccination and secondary prevention strategies [1]. In 2022, an estimated 348,000 women died from CC globally; approximately 90% of these deaths occurred in low- and middle-income countries (LMICs) where access to preventive services remains limited [1]. Ghana exemplifies the significant challenges faced by many LMICs in tackling CC, ranking as the second most frequent cancer among Ghanaian women overall and among those aged 15–44 years [2]. Recent estimates indicate an age-standardized incidence rate of approximately 27 per 100,000 women and a mortality rate of 17–18 per 100,000 women [3]. Compounding the high incidence, a large proportion of CC cases in Ghana (approximately 70%) are diagnosed at an advanced stage, contributing significantly to the high mortality rates [1]. The Ghana National Reproductive Health Policy and Standards recommends cervical cancer screening using Visual Inspection with Acetic acid (VIA) and Pap tests for women of reproductive age (25–45 years); however, no funded national HPV-based program exists, and coverage remains <5% [4].

We searched Embase (OVID), LILACS, CINAHL (EBSCO), Scopus, MEDLINE (OVID), Theses Global (ProQuest), and Cochrane Trials (Wiley) from database inception until May 15, 2025 using the terms “Ghana”, “HPV”, “human papillomavirus”, “cervical cancer”, “cervical precancer”, and “screening”. Previous work in Ghana has consistently documented extremely low screening coverage, with only 2–4% of eligible women ever having been screened for cervical precancer [5,6]. Small-scale initiatives have demonstrated the feasibility of Visual Inspection with Acetic acid (VIA) and pilot HPV DNA testing strategies, often relying on multiple visits and centralized laboratories; however, none have reported a truly nationwide model integrated into routine general health services.

In response to the multifaceted challenges underlying poor CC screening uptake, particularly the critical gap in trained human resources in Ghana, the Cervical Cancer Prevention and Training Centre (CCPTC) was established in 2017 at the Catholic Hospital in Battor, Ghana [7]. Over the years, the CCPTC has provided hands-on training to diverse cadres of health workers (HWs) in various screening techniques (VIA, Pap sampling, HPV testing, and basic mobile colposcopy) and the treatment of precancerous lesions using methods suitable for low-resource settings, such as thermal ablation and cryotherapy [7]. Recognizing prevailing challenges such as loss to follow-up, the CCPTC has implemented screening campaigns based on concurrent double [8] and triple [9] testing approaches, both seeking to minimize loss of patients to follow-up and treatment by reducing health facility visits. Building directly upon this foundation, the Rotary ‘Protect Your Pearl’ initiative (PYPI) was launched as a large-scale initiative aimed at further demonstrating the feasibility of scaling up integrated CC prevention services across Ghana [10]. The project was funded by a Rotary Foundation global grant, which covered the procurement of thermal coagulators that were distributed to CCPTC-trained teams across all 16 regions [10]. The PYPI aimed to screen approximately 1,600 women nationwide between January and February 2025, leveraging the CCPTC alumni network and the newly distributed equipment to offer screening services.

Documenting and analyzing the implementation process and outcomes of this project can provide invaluable evidence to inform policy, guide program design, and contribute to the development of scalable and sustainable solutions for CC prevention in low-resource countries striving to meet the WHO elimination (90-70-90) [11] targets, and for the broader global health community seeking effective pathways to close implementation gaps. This paper aims to report the initial outcomes of the Rotary ‘Protect Your Pearl’ cervical precancer screening and treatment project. Specifically, we detail the project’s operational framework, geographical reach (across Ghana’s 16 regions), prevalence of screening outcomes using standard methods, and rates of treatment completion among eligible women identified through the CCPTC-supported network. Our secondary objectives were to examine factors associated with screening outcomes among women who participated in this nationwide initiative.

## Methods

### Study design, setting, and participant eligibility

This cross-sectional study evaluates the feasibility of implementing a large-scale cervical precancer screening program based on concurrent hr-HPV testing and direct visual inspection in Ghana. The data were collected during the operational period of the initiative, which spanned from January 15 to February 22, 2025. The reporting of this study adheres to the Strengthening the Reporting of Observational Studies in Epidemiology (STROBE) guidelines for cross-sectional studies. Facility-based opportunistic screening and treatment activities were conducted at 29 designated health facilities, encompassing a variety of governmental and private healthcare facilities, operating at different levels of service delivery (S1 Fig). This diverse range of sites was chosen to reflect the varied healthcare delivery landscape within Ghana and enhance the generalizability of our findings to other contexts.

Eligible participants were asymptomatic women 25 years and older invited to attend the designated screening sites during the project period. The exclusion criteria included current pregnancy, a history of total hysterectomy, and current treatment for CC. Women presenting with symptoms suggestive of invasive CC (e.g., unexplained vaginal bleeding, pelvic pain) were not enrolled in this screening pathway but were instead directed to appropriate diagnostic and clinical management protocols for symptomatic individuals.

### Facility selection, project organisation, implementation, and oversight

The 29 facilities were purposively selected on the basis of (i) presence of at least one CCPTC-trained alumnus capable of unsupervised screening and (ii) geographic representation across all 16 regions and levels of care (CHPS compounds to regional hospitals), mirroring the expanded hub-and-spoke model previously described [12].

The initiative was conceived to screen about 1,600 women for cervical precancer using HPV DNA testing and VIA, provide thermal ablation for eligible lesions, raise public awareness about CC prevention, and promote early detection strategies [10]. The project was a multi-stakeholder partnership, with key collaborators being the Rotary Club of Accra-East (Ghana) and the Rotary Club of Eau Claire (Wisconsin, USA), who were the primary initiators and secured funding through The Rotary Foundation; the CCPTC at Catholic Hospital, Battor, which served as the lead technical partner for HW training, quality assurance, and logistical coordination of equipment distribution; Sansure Biotech Inc., who provided reagents for HPV testing and Omni Diagnostics, a local partner involved in the diagnostics supply chain. The Screen-and-Treat Research Group, Centre for Research in Applied Biology, Sunyani, provided research support. The initiative’s mobilization and awareness efforts were also supported by an extensive network of 53 Rotary Clubs, 3 Rotaract Clubs, and one Interact Club across Ghana [10].

A central coordinating committee, comprising representatives from the lead Rotary Clubs and CCPTC, was established to oversee the strategic planning, overall management, and execution of the initiative. The CCPTC played a pivotal role in guiding the technical aspects, including the standardized training of all participating HWs and the strategic distribution of thermal coagulator devices to the 29 selected screening sites across all 16 regions of Ghana. The screening components of the project were based on an expanded hub-and-spoke model for operational coordination and technical support, with the CCPTC serving as the central ‘hub,’ providing expertise, training, and ongoing mentorship, while the 29 screening sites functioned as the ‘spokes,’ delivering services within their communities [12]. This approach leveraged the existing strengths and reputation of CCPTC, facilitating a more sustainable and efficient scale-up of services compared to creating entirely new parallel systems. The central coordinating team regularly monitored key process indicators from the field data, such as the number of women screened per site, HPV and VIA ‘positivity’ rates, and treatment rates.

### Health worker training, recruitment, and ‘telementoring’ as a means of quality assurance

The HWs involved in the PYPI were primarily graduates of the comprehensive double-module training program developed, hosted, and delivered by the CCPTC. Components of this training program (which last four weeks in total) have been described in details elsewhere [13,14]. In brief, Module 1 focuses on foundational knowledge and skills essential for establishing and running a cervical screening service, with emphasis on the administrative and logistical aspects of screening programs. Module 2 builds on the competencies acquired in Module 1, providing advanced training in VIA and mobile colposcopy. A significant part of Module 2 is equipping trainees with the knowledge and practical skills to treat cervical precancerous lesions using ablative methods, primarily thermal ablation, which was the method employed in the PYPI due to the distributed equipment. While graduates of the training program have been involved in screening in other nationwide programs of this nature [15], this was the first time in which an extended range of cervical precancer screening services were available to women, since all HWs involved were equipped with the skill and equipment to treat eligible cervical precancers.

Trainees and alumni of CCPTC are typically integrated into professional WhatsApp groups moderated by CCPTC trainers, in order to facilitate peer-to-peer learning, rapid consultation on clinical queries, dissemination of updated information, and continuous professional development. To ensure sustained quality of screening and treatment services across the 29 geographically dispersed sites, this system of remote mentoring (termed ‘*telementoring*’) was used for ongoing QA in the PYPI, leveraging the CCPTC’s established ‘hub-and-spoke’ model of support. HWs were encouraged to share anonymized images from mobile colposcopes or mobile phones (if used for VIA documentation or difficult cases) and discuss challenging clinical scenarios with a gynecologist and experienced trainers at CCPTC via secure channels.

### Cervical specimen collection and VIA

During cervical screening sessions, women first received comprehensive counseling about the procedure’s benefits, potential risks, and possible results. In the dorsal lithotomy position, a speculum was used to visualize the cervix, and a cervical sample was collected for HPV DNA testing using a sterile swab stick (Bioline Diagnostics LLP, New Delhi, India). This involved rubbing the swab stick in a circular motion on the ectocervix and inserting the brush or swab into the cervical os and rotating it 360 degrees to collect cells from the transformation zone (TZ). The dry swab samples were sealed in a plain tube. These samples were typically sent to the central laboratory of the CCPTC within 1 week. If delayed, they were stored refrigerated (0–4°C) or frozen (−16°C) at the collection site and submitted within two weeks. Before testing, the samples were kept frozen (−16°C).

Following HPV DNA sample collection, VIA was performed as per standard procedure [16]. In brief, 5% acetic acid was applied to the cervix, and examined after 120 seconds under good lighting for the presence of acetowhite lesions in the TZ (which would indicate a ‘positive’ result).

### Transformation zone classification

During the VIA procedure, HWs assessed and classified the cervical TZ according to the 2011 International Federation for Cervical Pathology and Colposcopy (IFCPC) nomenclature [17,18], as a key determinant for eligibility for thermal ablation. The TZ types were recorded as:

***Type 1***: The entire TZ, including the entire squamocolumnar junction (SCJ), is ectocervical and therefore fully visible.

***Type 2***: The TZ has an endocervical component, but the entire SCJ is still fully visible, either directly or with gentle manipulation using a cotton-tipped applicator or an endocervical speculum if available.

***Type 3***: The TZ extends into the endocervical canal such that the upper limit of the SCJ is not fully visible, even with manipulation.

### Laboratory processing of submitted cervical specimens and HPV DNA testing with the MA-6000 platform

At the central laboratory, the cervical specimens were preprocessed and tested for HPV DNA using HPV 13 + 2 DNA diagnostic kits (S3057E; Sansure Biotech Inc., Hunan, China) on an MA-6000 real-time PCR device (Sansure Biotech Inc.). All tests were run in strict accordance with the manufacturer’s instructions [19], and as described elsewhere [20]. For full genotyping, samples were processed in batches of up to 24, with the platform operating in full quantitative mode. This allowed for the detection and differentiation of the following high-risk HPV genotypes: 16, 18, 31, 33, 35, 39, 45, 51, 52, 53, 56, 58, 59, 66, and 68. Test results were typically available within approximately two hours.

### General strategy for management of screen positives, follow-up, and referral pathways

Women were deemed eligible for immediate on-site treatment with thermal ablation if they were VIA positive and the entire TZ was fully visible (IFCPC TZ type 1 or 2), ensuring the full extent of the SCJ and any lesion could be assessed and reached by the ablation probe; the lesion was confined to the ectocervix, did not extend into the endocervical canal beyond the reach of the probe, did not extend onto the vaginal walls, and covered less than 75% of the cervical surface; and there was no clinical suspicion of invasive CC or glandular disease (e.g., adenocarcinoma in situ) [21]. The thermal ablation procedure involved the application of a pre-heated metallic probe (probe types such as 16 mm or 19 mm flat or nipple probes were available, selected based on cervical anatomy and lesion characteristics directly) to the abnormal cervical tissue within the TZ. Treatment was typically administered at a probe temperature of 100°C for 40 seconds per application, as recommended by WHO guidelines [21]. Multiple, slightly overlapping applications were performed as necessary to ensure complete ablation of the entire TZ, including the lesion and a small margin of surrounding normal tissue. The procedure was generally performed without anesthesia, though local anesthetic could be considered if available and deemed necessary by the provider.

HPV results were communicated to participants and providers within two weeks via telephone or in-person return visits arranged by the screening site. HPV-positive,VIA-negative women were advised to undergo HPV DNA testing again after 1 year; they were also given the choice of retesting at a nearby facility or at the ‘spoke’ where they were initially screened. HPV-negative women were reassured and advised to return for routine screening in 5 years (if VIA negative) or in 6 months to 1 year (if minor changes were found at VIA). HPV negative, VIA negative women who were HIV positive were counselled to be rescreened in 3 years. Women with major cervical lesions who were ineligible for on-site thermal ablation were counseled and referred for further diagnostic evaluation and appropriate management (e.g., LEEP) at a higher-level facility.

### Ethical considerations

The study complied with the Declaration of Helsinki (1964) and its later amendments. Written informed consent was sought from all women prior to registration, interviews, sample collection, and cervical screening. The study was approved by the Committee for Human Research and Ethics, University of Energy and Natural Resources, Sunyani, Ghana (ref. CHRE/CA/363/025).

### Sample size

This study comprised all women screened by CCPTC graduates as part of the Rotary PYPI. We did not perform an *a priori* sample size estimation because the study was embedded within a service-delivery initiative funded by a fixed Rotary Foundation grant. Thus, realised sample of 1,636 women was determined by the availability of funds/resources in the initiative instead of research-driven statistical requirements.

### Data collection and transmission

Standardized paper-based case report forms (CRFs) in routine use at the CCPTC for clinical data collection were used for the PYPI consistently across all 29 screening sites. These CRFs were developed to capture all essential demographic, clinical, screening, and treatment variables. After providing education and counseling and obtaining written consent, data were collected by HWs and subsequently sent by courier to the CCPTC, where the data were transcribed into REDCap version 11·0·3 (Vanderbilt University, Nashville, TN, USA) [22] and stored securely within databases hosted and managed by the CCPTC. Prior to the analyses reported here, the databases were queried, and data were extracted and converted into an Excel spreadsheet (S2 File), followed by manual cross-checking for accuracy.

### Study variables and outcomes

Data captured in the CRFs included sociodemographic information, behavioral and lifestyle factors, clinical history, HPV specimen information, VIA findings, adequacy of examination, and treatment information (if applicable). HPV test results, including the overall hr-HPV status and specific genotype(s) identified, were recorded once testing was completed at the CCPTC. The primary screening outcomes were rates of hr-HPV infection and of cervical lesions observed at VIA. Secondary outcomes specific to the PYPI were referral efficiency, defined as the effectiveness of the referral system between satellite centers and the central laboratory for cases requiring advanced diagnostic testing or treatment and treatment rates, defined as the proportion of women who received follow-up care, including those treated with procedures like thermal ablation or loop electrosurgical excision procedure.

### Statistical methods

Continuous variables were inspected for distributions using histograms. Normally distributed continuous variables are summarized descriptively as means with standard deviations (SDs), while skewed variables are summarized as medians with interquartile ranges (IQRs). Categorical data are reported as frequencies and percentages. The overall prevalence of hr-HPV and cervical lesions (VIA ‘positivity’) are expressed as proportions, accompanied by their 95% Clopper–Pearson confidence intervals (CIs). The prevalence of hr-HPV was further disaggregated by region, specific hr-HPV genotype (and grouping based on the IARC’s classification [23]), and whether infections involved single or multiple genotypes. We fit separate univariable and multivariable logistic regression models to identify factors potentially associated with hr-HPV infection and VIA ‘positivity’. Both multivariable models were fitted using the backward stepwise elimination procedure with a liberal probability cut-off of 0·25 for variable selection. Given the minimal amount of missing data across the assessed variables, participants with missing values were listwise-deleted from the regression analyses. Effect estimates and directionality of the observed associations are reported as odds ratios and adjusted odds ratios (aORs), each with its corresponding 95% CI. All statistical analyses were performed using Stata release 17·0 (StataCorp LLC, College Station, TX, USA). Hypothesis tests were considered statistically significant at a two-tailed alpha level of 5%.

## Results

### Participant selection and dispositions

**Fig 1:**
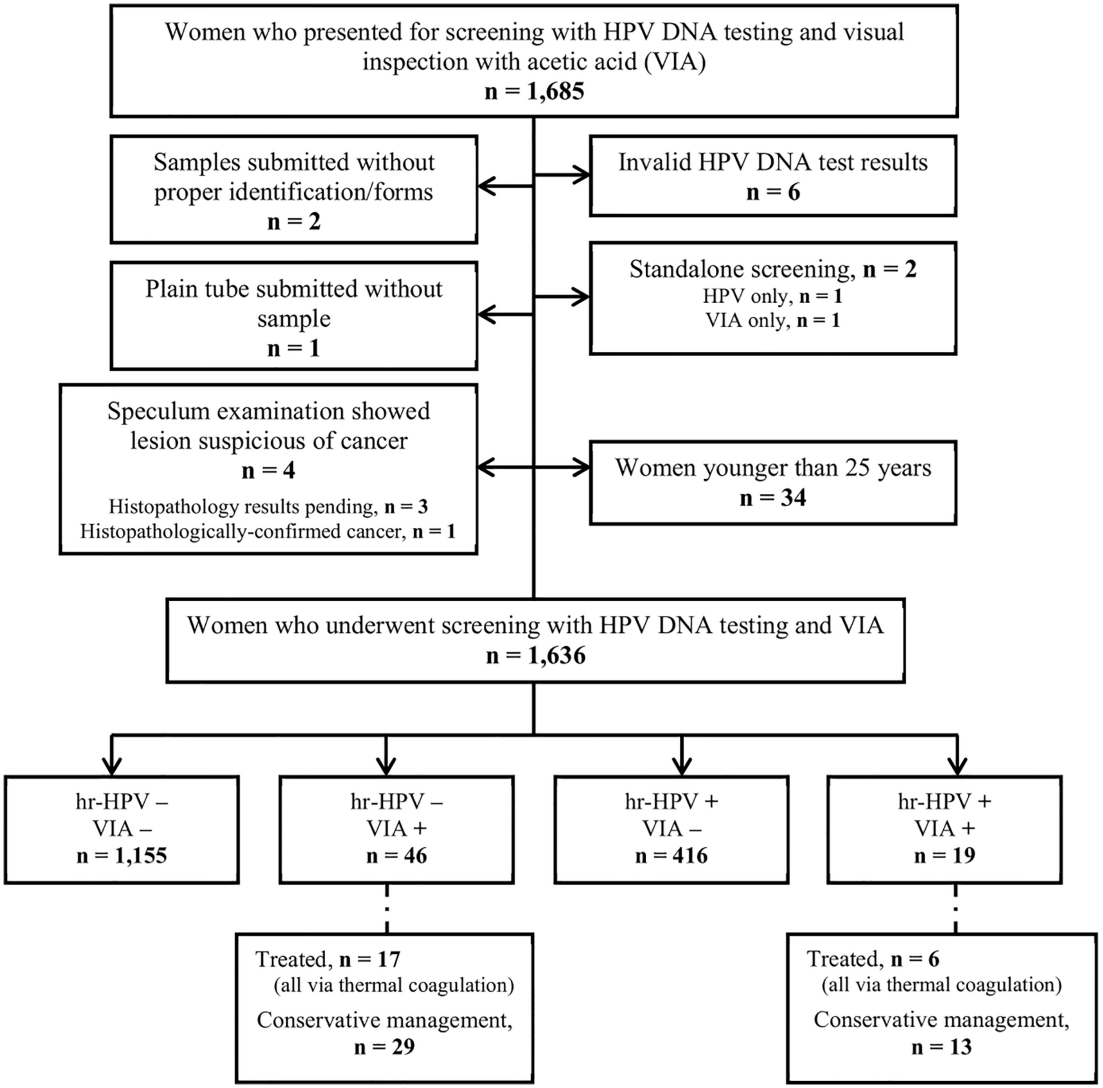
Flow diagram illustrating participant enrollment, screening with HPV DNA testing and VIA, and subsequent management in the Rotary ‘Protect Your Pearl’ Initiative.

A total of 1,685 women presented for cervical precancer screening (Fig 1), among whom 1,636 underwent and had valid results for both HPV DNA testing and VIA, and were included in the present analyses. 435 women (26·6%) tested positive for hr-HPV; among these, 416 (95·6% of hr-HPV positives; 25·4% of the total screened) were hr-HPV positive and VIA negative. Nineteen women (4·4% of hr-HPV positives; 1·2% of the total screened) were positive for both hr-HPV and VIA. Among hr-HPV + /VIA+ women, 6 (31·6%) were treated (all via thermal coagulation) and 13 (68·4%) were managed conservatively.

The sociodemographic and clinical details of women screened in the PYPI are shown in Table 1. The mean age was 38·5 (SD, 10·4) years, a greater proportion were married or cohabiting (n = 1,089, 66·6%), and the median parity was 2 (IQR: 1, 3). Regarding clinical history, 1·2% (n = 20) reported being HIV positive, while the HIV status was unknown or missing for 595 (36·4%). Prior precancer screening was reported among 322 (19·7%) women.

**Table 1:** Sociodemographic and clinical details of the 1,636 women screened using HPV DNA testing and VIA under the Rotary ‘Protect Your Pearl’ Initiative.

Variable	Estimate
Age, years; mean (SD)	38·8 (10·4)
Age group, years; n (%)	
25–34	658 (40·2)
35–44	553 (33·8)
45–54	283 (17·3)
≥55	141 (8·6)
Missing	1 (0·1)
Marital status	
Single	306 (18·7)
Has a steady partner	91 (5·6)
Married/cohabiting	1,089 (66·6)
Divorced	63 (3·8)
Widowed	82 (5·0)
Missing	5 (0·3)
Number of children, median (IQR)	2 (1, 3)
Education, n (%)	
No schooling	128 (7·8)
Primary	108 (6·6)
Junior high school	266 (16·3)
Senior high school	215 (13·1)
Tertiary	904 (55·3)
Other	10 (0·6)
Missing	5 (0·3)
Religious affiliation, n (%)	
Christianity	1,421 (86·9)
Islamic	204 (12·5)
African traditional religion	8 (0·5)
Other	1 (0·1)
Missing	2 (0·1)
NHIS coverage, n (%)	1204 (73·6)
Ever smoked, n (%)	1 (0·1)
HIV status, n (%)	
Positive	20 (1·2)
Negative	1,021 (62·4)
Unknown/missing	595 (36·4)
Hypertension, n (%)	203 (12·4)
Sickle cell disease, n (%)	17 (1·0)
Diabetes, n (%)	45 (2·8)
Asthma, n (%)	31 (1·9)
History of contraceptive use, n (%)	794 (48·5)
Prior cervical (pre)cancer screening, n (%)	322 (19·7)

HIV, human immunodeficiency virus; HPV, human papillomavirus; IQR, interquartile range; NHIS, National Health Insurance Scheme; SD, standard deviation; VIA, Visual Inspection with Acetic acid.

### Screening characteristics and outcomes among the study participants

Gross cervical inspection revealed abnormality in 19 (1·2%) participants. The commonest TZ type was type 3 (68·9%), followed by type 2 (22·2%) and type 1 (8·4%). The VIA ‘positivity’ rate was 4·0% (95% CI, 3·1–5·0) (Table 2). The overall hr-HPV prevalence was 26·6% (95% CI, 24·5–28·8). HPV52 was the most prevalent at 5·3% (95% CI, 4·3–6·5), followed by HPV58 (4·4%, 95% CI, 3·5–5·5) and HPV51 (3·6%, 95% CI, 2·8–4·6). HPV35 was detected in 2·7% (95% CI, 2·0–3·6), HPV18 in 2·4% (95% CI, 1·8–3·3), HPV31 in 2·5% (95% CI, 1·9–3·4), HPV56 in 2·4% (95% CI, 1·8–3·3), and HPV16 in 1·8% (95% CI, 1·3–2·6). Co-infection with high-risk and probable high-risk HPV types was observed in 2·0% (95% CI, 1·4–2·8) of women (Table 2).

**Table 2:** Gross clinical characteristics and laboratory outcomes (including HPV genotype distributions) recorded among the 1,636 women screened using HPV DNA testing and VIA under the Rotary ‘Protect Your Pearl’ Initiative.

Abnormal gross vulval inspection findings, n (%)	13 (1·0)
Missing	2 (0·1)
Abnormal vulval inspection findings, n = 13, n (%)	
Warts	5 (38·5)
Others^β^	8 (61·5)
Abnormal gross vaginal inspection findings, n (%)	3 (0·2)
Missing	11 (0·7)
Abnormal vaginal inspection findings, n = 3, n (%)	
Warts	3 (100·0)
Gross cervical inspection findings, n (%)	
Normal	1,611 (98·5)
Abnormal	19 (1·2)
Missing	6 (0·3)
Abnormal cervical inspection findings, n = 19, n (%)	
Leukoplakia	3 (15·8)
Polyps	9 (37·4)
Others	5 (26·3)
Missing	2 (10·5)
TZ type on VIA, n (%)	
1	138 (8·4)
2	363 (22·2)
3	1,127 (68·9)
Missing	8 (0·5)
VIA ‘positive’, % (95% CI)	4·0 (3·1–5·0)
High-risk HPV, % (95% CI)	
16	1·8 (1·3–2·6)
18	2·4 (1·8–3·3)
31	2·5 (1·9–3·4)
33	0·5 (0·2–1·0)
35	2·7 (2·0–3·6)
39	1·5 (1·0–2·3)
45	1·1 (0·6–1·6)
51	3·6 (2·8–4·6)
52	5·3 (4·3–6·5)
56	2·4 (1·8–3·3)
58	4·4 (3·5–5·5)
59	1·5 (1·0–2·3)
Probable high-risk HPV, % (95% CI)	
66	2·1 (1·5–2·9)
68	2·3 (1·6–3·1)
Potential high-risk HPV, % (95% CI)	
53	3·5 (2·7–4·5)
High risk HPV + , % (95% CI)	26·6 (24·5–28·8)
Single high-risk HPV + , % (95% CI)	19·2 (17·4–21·2)
Multiple high-risk HPV + , % (95% CI)	7·4 (6·2–8·8)
High risk AND probable HPV + , % (95% CI)	2·0 (1·4–2·8)
High risk AND probable HPV+ AND potential HPV + , % (95% CI)	0·2 (0·1–0·6)

CI, confidence interval; HPV, human papillomavirus; TZ, transformation zone; VIA, Visual Inspection with Acetic acid.

β Includes ulcerations, razor bumps, inflammation, clitoral tag, painless massive swelling of the right labia, and labia majora not seen.

### Factors associated with hr-HPV infection and VIA ‘positivity’

In the multivariable model, although based on a small number of HIV-positive women (n = 20), HIV infection was associated with substantially higher odds of hr-HPV infection (aOR=5·77; 95% CI, 2·07–16·13, P = 0.001). Compared to single women, those with a steady partner had more than twice the odds of infection (aOR=2·02; 95% CI, 1·22–3·36). In contrast, women who were married/cohabiting (aOR=0·51; 95% CI, 0·38–0·69) or widowed (aOR=0·43; 95% CI, 0·23–0·82) were independently associated with lower odds of hr-HPV infection (aOR 0.51 [95% CI 0.38–0.69], P < 0.001; aOR 0.43 [0.23–0.82], P = 0.011; and aOR 0.67 [0.48–0.92], P = 0.014) respectively. Further, women who had prior cancer screening showed significantly lower odds of infection compared to those who had never been screened (aOR=0·67; 95% CI, 0·48–0·92). Other variables, including religion, NHIS coverage, TZ type, and abnormal findings on gross vulval inspection, did not show independent associations with hr-HPV infection (Table 3).

**Table 3:** Exploratory logistic regression analysis of selected sociodemographic and clinical variables associated with high-risk HPV infection among the 1,636 women screened using HPV DNA testing and VIA under the Rotary ‘Protect Your Pearl’ Initiative.

Characteristic	Univariable analyses		Multivariable analysis	
	OR (95% CI)	*p*-value	aOR (95% CI)	*p*-value
Age group, years				
25–34	1·37 (0·91–2·07)	0·137	–	–
35–44	0·92 (0·60–1·41)	0·715	–	–
45–54	0·70 (0·44–1·14)	0·151	–	–
≥55 (ref.)	1·00	–	–	–
Marital status				
Single (ref.)	1·00	–	1·00	–
Has a steady partner	2·17 (1·35–3·49)	0·001*	2·02 (1·22–3·36)	0·006*
Married/cohabiting	0·49 (0·37–0·64)	<0·001*	0·51 (0·38–0·69)	<0·001*
Divorced	0·77 (0·43–1·38)	0·381	0·67 (0·35–1·27)	0·216
Widowed	0·54 (0·31–0·94)	0·031*	0·43 (0·23–0·82)	0·011*
Number of children				
0	2·11 (1·39–3·19)	<0·001*	–	–
1–2	1·34 (0·89–2·01)	0·165	–	–
3–4	1·11 (0·74–1·67)	0·619	–	–
≥5 (ref.)	1·00	–	–	–
Educational level				
No schooling (ref.)	1·00	–	–	–
Primary	0·86 (0·46–1·63)	0·647	–	–
Junior high school	1·77 (1·08–2·88)	0·023*	–	–
Senior high school	1·45 (0·87–2·42)	0·157	–	–
Tertiary	1·23 (0·79–1·91)	0·379	–	–
Other	1·53 (0·37–6·31)	0·556	–	–
Religion				
Christianity	1·14 (0·23–5·67)	0·874	1·22 (0·22–6·89)	0·822
Islamic	0·75 (0·15–3·88)	0·736	0·83 (0·14–4·89)	0·840
African traditional religion (ref.)	1·00	–	1·00	–
Past contraceptive use (ref. = ‘no’)	1·02 (0·82–1·27)	0·881	–	–
Prior (pre)cancer screening (ref. = ‘no’)	0·66 (0·49–0·89)	0·006*	0·67 (0·48–0·92)	0·014*
NHIS coverage (ref. = ‘no’)	1·18 (0·91–1·52)	0·211	1·22 (0·93–1·61)	0·162
HIV status (ref. = ‘negative’)				
Positive	5·17 (2·04–13·08)	0·001*	5·77 (2·07–16·13)	0·001*
Unknown	0·89 (0·69–1·13)	0·336	0·88 (0·67–1·14)	0·323
Hypertension (ref. = ‘no’)	0·71 (0·50–1·02)	0·063	–	–
Sickle cell disease (ref. = ‘no’)	0·59 (0·17–2·06)	0·407	–	–
Diabetes (ref. = ‘no’)	1·13 (0·59–2·16)	0·723	–	–
Asthma (ref. = ‘no’)	0·80 (0·34–1·88)	0·611	–	–
Abnormal finding on gross vulval inspection	0·50 (0·11–2·27)	0·369	0·29 (0·04–2·35)	0·245
Abnormal finding on gross vaginal inspection	1·39 (0·13–15·32)	0·790	–	–
Abnormal finding on gross cervical inspection	0·52 (0·15–1·79)	0·298	–	–
TZ type				
1	1·19 (0·80–1·76)	0·386	1·22 (0·22–6·89)	0·209
2	1·20 (0·92–1·56)	0·171	1·31 (0·98–1·74)	0·070
3 (ref.)	1·00	–	1·00	–

aOR, adjusted odds ratio; CI, confidence interval; HIV, human immunodeficiency virus; NHIS, National Health Insurance Scheme; OR, odds ratio; ref., reference category; TZ, transformation zone.

In the multivariable logistic regression (Table 4), HIV-positive status was significantly associated with higher odds of VIA ‘positivity’ (aOR=7.49; 95% CI, 1.99–28.19, P = 0.003). The wide confidence interval signals limited statistical precision and warrants cautious interpretation pending larger studies. In addition, women with a type 2 TZ had significantly increased odds of VIA positivity compared to those with a type 3 TZ (aOR=2.48; 95% CI, 1.43–4.31).

**Table 4:** Exploratory logistic regression analysis of selected sociodemographic and clinical variables associated with VIA ‘positivity’ among the 1,636 women screened using HPV DNA testing and VIA under the Rotary ‘Protect Your Pearl’ Initiative.

Characteristic	Univariable analyses		Multivariable analysis	
	OR (95% CI)	*p*-value	aOR (95% CI)	*p*-value
Age group, years				
25–34	1·25 (0·48–3·30)	0·646	–	–
35–44	1·07 (0·48–2·90)	0·888	–	–
45–54	0·99 (0·33–2·97)	0·995	–	–
≥55 (ref.)	1·00	–	–	–
Marital status				
Single (ref.)	1·00	–	–	–
Has a steady partner	1·52 (0·46–5·05)	0·497	–	–
Married/cohabiting	1·49 (0·72–3·07)	0·282	–	–
Divorced	0·53 (0·07–4·28)	0·553	–	–
Widowed	1·69 (0·51–5·64)	0·392	–	–
Number of children				
0 (ref.)	1·00	–	–	–
1–2	1·07 (0·52–2·20)	0·845	–	–
3–4	1·15 (0·57–2·32)	0·705	–	–
≥5	1·98 (0·88–4·42)	0·097	–	–
Education				
No schooling (ref.)	1·00	–	–	–
Primary	1·50 (0·39–5·75)	0·550	–	–
Junior high school	2·11 (0·70–6·42)	0·186	–	–
Senior high school	1·04 (0·30–3·63)	0·947	–	–
Tertiary	1·10 (0·38–3·17)	0·859	–	–
Other	3·44 (0·35–34·13)	0·291	–	–
Religion				
Christianity	1·20 (0·54–2·66)	0·658	–	–
Islamic (ref.)	1·00	–	–	–
Past contraceptive use (ref. = ‘no’)	1·16 (0·71–1·90)	0·561	–	–
Prior (pre)cancer screening (ref. = ‘no’)	0·92 (0·48–1·73)	0·793	–	–
NHIS coverage (ref. = ‘no’)	0·93 (0·54–1·63)	0·810	–	–
HIV status (ref. = ‘negative’)				
Positive	4·69 (1·32–16·72)	0·017*	7·49 (1·99–28·19)	0·003*
Unknown	1·20 (0·70–2·06)	0·504	1·24 (0·71–2·14)	0·447
Hypertension (ref. = ‘no’)	1·14 (0·56–2·34)	0·720	–	–
Sickle cell disease (ref. = ‘no’)	1·52 (0·19–11·63)	0·688	–	–
Diabetes (ref. = ‘no’)	0·54 (0·07–3·99)	0·548	–	–
Abnormal finding on gross cervical inspection	2·89·81 (0·65–12·78)	0·162	3·47 (0·74–16·26)	0·114
TZ type				
1	1·21 (0·46–3·14)	0·698	1·21 (0·45–3·21)	0·705
2	2·48 (1·47–4·19)	0·001*	2·48 (1·43–4·31)	0·001*
3 (ref.)	1·00	–	1·00	–

aOR, adjusted odds ratio; HPV, human papillomavirus; OR, odds ratio; NHIS, National Health Insurance Scheme; ref., reference category; TZ, transformation zone; VIA, Visual Inspection with Acetic acid.

### Regional distributions of hr-HPV prevalence

**Fig 2:**
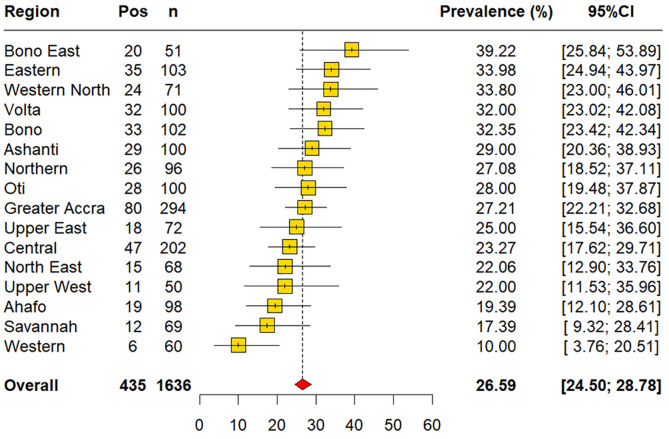
Forest plot showing the distribution of hr-HPV prevalence stratified by region among women screened under the Rotary ‘Protect Your Pearl’ Initiative. n, number of women screened; CI, confidence interval.

We observed substantial variability in prevalence across the 16 regions of Ghana (Fig 2). Bono East region showed the highest prevalence at 39·2% (95% CI, 25·8–53·9), followed by Eastern region (34·0%; 95% CI, 24·9–44·0) and Western North region (33·8%; 95% CI, 23·0–46·0). Western region showed the lowest prevalence at 10·0% (95% CI, 3·8–20·5), with Savanna region and Ahafo region following closely with relatively low prevalence estimates of 17·4% (95% CI, 9·3–28·4) and 19·4% (95% CI, 12·1–28·6), respectively.

## Discussion

This study reports on research outcomes of the Rotary PYPI, a large-scale CC screening program conducted across all 16 regions of Ghana. A major strength of the PYPI was that it strategically leveraged the existing infrastructure and expertise of the CCPTC, along with the procurement and distribution of 50 thermal coagulator devices through the Rotary grant, which directly addressed a critical equipment gap and enabled point-of-care treatment. The multi-stakeholder collaboration involving Rotary Clubs (local and international), CCPTC as the technical lead, industry partners, and numerous local Rotary/Rotaract/Interact clubs was instrumental in mobilizing resources, raising awareness, and coordinating implementation. The operational framework of the PYPI successfully translated into tangible public health action, reaching 1,685 women across all regions for both screening and analysis. We found a substantial underlying hr-HPV burden of 26·6% and a 4·0% VIA ‘positivity’ rate. From an implementation perspective, 35·4% (23 out of 65) of all VIA-positive women ultimately received onsite treatment, while the remaining were managed conservatively.

The observed hr-HPV prevalence of 26·6% (95% CI, 24·5–28·8) aligns with some previous reports from Ghana, such as 37·2% in Kumasi [24] and 25·5% among pregnant and postnatal women in Battor [25], and another more recent nationwide estimate of 29·1% [15]. When compared regionally and continentally, our prevalence is higher than the estimated prevalence in women with normal cytology in Africa (around 22%) [26] but falls below the pooled estimate for sub-Saharan Africa (34%), which includes women with varying cytology statuses [27]. The relatively high educational attainment within this cohort (55·3% with tertiary education) makes the observed 26·6% hr-HPV prevalence particularly concerning. Higher education levels often correlate with increased health awareness and lower HPV infection risk [28–30]. Finding such a high prevalence in this group suggests that the underlying hr-HPV burden in the broader Ghanaian population, particularly among women with lower educational attainment or facing greater socioeconomic barriers, might be even higher. Further, the strong association between HIV infection and hr-HPV infection (aOR=5·77) is consistent with extensive literature documenting the synergistic relationship between these two viruses, where immunosuppression hinders HPV clearance [31]. This highlights women living with HIV as a critical sub-population requiring intensified and tailored screening strategies. However, the large proportion of participants with unknown or missing HIV status (36·4%) significantly limited our ability to fully quantify the contribution of HIV to the overall hr-HPV burden in this cohort and represents a missed opportunity for integrating HIV services with HPV screening.

Another striking finding of this study is the hr-HPV genotype distribution, characterized by the predominance of HPV52, HPV58, HPV51, and HPV35. While global data suggest that HPV16 and HPV18 account for approximately 70% of CC cases [31,32] our findings resonate more closely with patterns observed in some other Ghanaian studies that report a higher prevalence and diversity of non-16/18 hr-HPV types. One Ghanaian study reported HPV56, 52, and 18 as the most common [24], and another noted HPV16, 58, 18, 35, and 52 as common in women with normal cytology, and HPV16, 18, 45, 59, and 35 in CC cases [33]. A meta-analysis suggested HPV16 and HPV35 are key genotypes in West Africa [27]; however, the particularly low prevalence of HPV16 found in our large, nationwide cohort is noteworthy and differs even from some Ghanaian studies. This unique genotype distribution has important implications for HPV vaccination strategies in Ghana. Currently available bivalent and quadrivalent vaccines (targeting HPV16/18) would offer limited direct protection against the predominant non-vaccine hr-HPV types in this nationwide cohort [33]. On the other hand, both bivalent and quadrivalent vaccines have been found to confer some partial cross-protective immunity against non-vaccine HPV types that are phylogenetically related to the vaccine targets, most consistently HPV31, 33, and 45 [34,35]. This cross-protection is generally less robust and may wane over time compared to the direct protection against HPV16 and 18 [35]. While the nonavalent vaccine provides broader coverage by including HPV31, 33, 45, 52, and 58 [36], it would still miss HPV51 and 35, which were among the top four most prevalent types identified here. The observed dominance of types like HPV52, 58, 51, and 35 requires further investigation, but may reflect unique local transmission dynamics influenced by sexual networks or behavioral factors, population-specific host genetic susceptibilities, interactions with co-endemic infections altering immune responses, or potentially indicate that these genotypes exhibit higher persistence rates within the Ghanaian population compared to HPV16/18.

### Strengths and limitations

To our best knowledge, this is the first time a non-targeted nationwide cervical screening intervention in Ghana has been systematically evaluated and documented. This study has several notable strengths. Its large-scale and nationwide reach, encompassing all 16 regions of Ghana, provides valuable data on the implementation of cervical screening across diverse settings within the country. The use of hr-HPV DNA testing aligns with current international best practices and WHO recommendations, and relying on HWs trained through the standardized, comprehensive CCPTC curriculum likely enhanced the consistency of screening procedures compared to programs with less structured training. Although the model required central laboratory HPV testing and thermal-ablation equipment, it leveraged existing CCPTC alumni infrastructure and ubiquitous mobile technology for telementoring, rendering it more feasible for scale-up than parallel vertical programmes. Ghana’s current opportunistic VIA/Pap strategy can be incrementally strengthened by embedding this alumni-driven concurrent-testing approach within routine services, thereby addressing the critical human-resource and equipment gaps identified in the 2014 policy [4].

Together with prior small-scale studies, our findings support a shift toward HPV screening complemented by VIA triage in resource-limited settings. The high prevalence of diverse hr-HPV types highlights the need for broad-coverage HPV tests and eventual adoption of vaccines covering non-16/18 genotypes. The demonstrated feasibility of leveraging existing postal networks for specimen transport and mobile telementoring for quality assurance provides a pragmatic blueprint for national programs aiming to meet WHO cervical cancer elimination targets. Policymakers should consider integrating this hub-and-spoke approach into routine health services, investing in decentralized laboratory capacity or sustained postal partnerships, and scaling up workforce training and digital mentorship to close the cervical cancer equity gap across sub-Saharan Africa.

However, the study also has several limitations. A major limitation is the lack of histological confirmation for the vast majority of participants; VIA ‘positivity’ was used as a subjective surrogate endpoint together with HPV DNA testing. Second, there is the potential for selection bias, as the participants were women who voluntarily presented for screening, who may differ from the general eligible population in Ghana, potentially being more health-conscious or educated, which could affect the generalizability of findings like hr-HPV prevalence. Third, we relied on self-reported data for variables such as HIV status, smoking history, and prior clinical history (which introduces potential recall or social desirability bias) and a high proportion of women had unknown HIV status. Fourth, despite standardized training, the inherent subjectivity of VIA interpretation and TZ classification could lead to inter-observer variability among HWs across the 29 sites [37], although telementoring aimed to mitigate this. Finally, the acknowledged absence of an *a priori* sample size calculation means that the statistical power for certain secondary analyses, such as subgroup comparisons or multivariable modeling, might be limited, although the overall sample size is substantial for a descriptive report of the screening program.

## Conclusions

The Rotary ‘Protect Your Pearl’ initiative demonstrates that a decentralised, alumni-led model integrating concurrent HPV genotyping, task-shifted VIA, and immediate thermal ablation is operationally feasible across Ghana’s 16 regions and can rapidly identify a substantial hr-HPV burden. The study revealed a high underlying burden of hr-HPV infection, characterized by a unique genotype profile with implications for current vaccine effectiveness. By minimising loss to follow-up through same-visit treatment and telementoring, this approach offers a pragmatic, resource-appropriate pathway to accelerate progress toward the WHO 90-70-90 elimination targets. With sustained investment in alumni training, decentralised laboratory capacity, and thermal-ablation devices, Ghana can transition from opportunistic to organised HPV-based screening while building sustainable local capacity. These results offer hope that even before a fully organized national screening program is in place, interim strategies can substantially reduce CC risk. The challenge now is to transition from a one-time campaign to a sustainable program that is embedded in Ghana’s health system, continuously quality-assured, and equitably accessible to women in all communities. Our findings will inform national policy deliberations and can serve as a blueprint for other countries embarking on the path to meet the WHO 90-70-90 targets for CC elimination.

## Supporting information

S1 Fig Map of Ghana showing the locations of the 29 facilities in which CCPTC trainees were stationed, and where women were screened in the Rotary ‘Protect Your Pearl’ Initiative (January–February 2025). (DOCX)

S2 File Raw data file. (XLSX)
