# Supplementary material for: A collaborative cervical precancer screening strategy with concurrent HPV genotyping and visual inspection using alumni of a training centre across Ghana: The Rotary ‘Protect Your Pearl’ initiative
Source: PLoS One. 2026 Jun 26;21(6):e0350573. doi: 10.1371/journal.pone.0350573 (PMC13309030; doi:10.1371/journal.pone.0350573)
Supplement: S1 Fig — (DOCX) [file pone.0350573.s001.docx]

**Supplementary Figure 1. Map of Ghana showing the locations of the 29 facilities in which CCPTC trainees were stationed, and where women were screened in the Rotary ‘Protect Your Pearl’ Initiative (January–February 2025).** The basemap and administrative boundaries were obtained from GADM maps and data (www.gadm.org). GADM data may be used to create maps for academic research publications, and the resulting maps can be published under a CC BY 4.0 license. The map was produced in QGIS by overlaying study-specific data on the GADM boundaries
